# Aztreonam Combinations with Avibactam, Relebactam, and Vaborbactam as Treatment for New Delhi Metallo-β-Lactamase-Producing Enterobacterales Infections—In Vitro Susceptibility Testing

**DOI:** 10.3390/ph17030383

**Published:** 2024-03-17

**Authors:** Małgorzata Brauncajs, Filip Bielec, Marlena Malinowska, Dorota Pastuszak-Lewandoska

**Affiliations:** 1 Department of Microbiology and Laboratory Medical Immunology, Medical University of Lodz, ul. Pomorska 251/C5, 92-213 Lodz, Poland; malgorzata.brauncajs@umed.lodz.pl (M.B.); marlena.malinowska@umed.lodz.pl (M.M.); dorota.pastuszak-lewandoska@umed.lodz.pl (D.P.-L.); 2 Medical Microbiology Laboratory, Central Teaching Hospital of Medical University of Lodz, ul. Pomorska 251/C5, 92-213 Lodz, Poland

**Keywords:** aztreonam, avibactam, vaborbactam, relebactam, antimicrobials, β-lactamase inhibitors, New Delhi metallo-β-lactamase, synergy

## Abstract

Antimicrobial resistance is a major global health issue. Metallo-β-lactamases (MBL), in particular, are problematic because they can inactivate all classes of β-lactams except aztreonam. Unfortunately, the latter may be simultaneously inactivated by serine β-lactamases. The most dangerous known MBL is New Delhi Metallo-β-lactamase (NDM). This study aimed to test the in vitro susceptibility to aztreonam in combination with novel β-lactamase inhibitors (avibactam, relebactam, and vaborbactam) in clinical strains of Enterobacterales NDM which is resistant to aztreonam. We investigated 21 NDM isolates—including *Klebsiella pneumoniae*, *Escherichia coli*, and *Citrobacter freundii*—which are simultaneously resistant to aztreonam, ceftazidime/avibactam, imipenem/relebactam, and meropenem/vaborbactam. MICs for aztreonam combinations with novel inhibitors were determined using the gradient strip superposition method. The most effective combination was aztreonam/avibactam, active in 80.95% strains, while combinations with relebactam and vaborbactam were effective in 61.90% and 47.62%, respectively. In three studied strains, none of the studied inhibitors restored aztreonam susceptibility. Aztreonam/avibactam has the most significant antimicrobial potential for NDM isolates. However, combinations with other inhibitors should not be rejected in advance because we identified strain susceptible only to tested combinations with inhibitors other than avibactam. Standardization committees should, as soon as possible, develop official methodology for antimicrobial susceptibility testing for aztreonam with β-lactamase inhibitors.

## 1. Introduction

Antimicrobial resistance is a major global health issue that leads to increased infection incidence, mortality, and healthcare costs associated with prolonged hospitalizations and last-resort treatment [1,2]. Just in 2019, about 1.27 million deaths were directly attributable to resistant pathogens [1]. One of the growing problems with antimicrobial resistance is carbapenem-resistant Gram-negative rods (*Acinetobacter* spp., *Pseudomonas* spp., and Enterobacterales order)—included with critical priority in the WHO priority list for the research and development of new antibiotics for antibiotic-resistant bacteria [2].

Carbapenemases are a diverse collection of enzymes capable of inactivating select antibiotics from the β-lactam group (e.g., penicillins, cephalosporins, carbapenems). In Poland, the most prevalent carbapenemases present in bacteria isolated from infections are within the Metallo-β-lactamases (MBL) family with the majority being the New Delhi MBL (NDM) [3,4]. MBLs, in particular, are problematic because they can inactivate all classes of β-lactams except monobactams (of which aztreonam is the only substance currently approved for use in humans) and amidinopenicillins (of which mecillinam is in use for the treatment of urinary tract infections). Additionally, MBLs are not inhibited by classic β-lactamase inhibitors (e.g., clavulanic acid, tazobactam, sulbactam), which makes their therapy very difficult [5,6,7,8]. Unfortunately, bacteria may possess more than one resistance mechanism simultaneously, making it quite possible to isolate Gram-negative MBL strains resistant also to aztreonam—inactivated by serine β-lactamases [9,10]. The ability to produce carbapenemases is of crucial clinical importance, as the genes encoding them can be potentially spread between bacteria of the same or different species through horizontal transfer via plasmids, integrons, or transposons [5].

β-lactamase inhibitors are substances used in combination with β-lactam antibiotics to counteract resistance to them by inhibiting serine β-lactamases (other than MBL). Novel β-lactamase inhibitors include avibactam, relebactam, and vaborbactam, which were approved for human use in 2015 and later. Their advantage over classic β-lactamase inhibitors is the ability to inhibit some extended-spectrum β-lactamases and carbapenemases [10,11].

Currently, the approved combinations of the novel β-lactamase inhibitors with antibiotics are ceftazidime/avibactam, imipenem/relebactam, and meropenem/vaborbactam. However, they do not show activity against all carbapenem-resistant strains. In the case of the previously mentioned MBL strains, we more often isolate strains that are also resistant to these drugs—e.g., in the cases of NDM variants [10,11].

One of the hopefully available solutions to this problem seems to be the combination of a novel β-lactamase inhibitor with aztreonam. In the literature, there are individual reports on the effectiveness of such combinations in vitro and in vivo in patients with resistant infections. However, only one study [12] simultaneously compared the effectiveness of avibactam, relebactam, and vaborbactam in restoring the susceptibility to aztreonam.

This study aimed to test the in vitro susceptibility to aztreonam in combination with novel β-lactamase inhibitors (avibactam, relebactam, and vaborbactam) in clinical strains of Enterobacterales producing NDM.

## 2. Results

NDM genes were present in a total of 21 of the studied strains (17 *Klebsiella pneumoniae*, three *Escherichia coli*, and one *Citrobacter freundii*), and 4 (*K. pneumoniae* only) of them were also positive with carbapenem-hydrolyzing *oxacillinase*, OXA-48. Three isolates (14%) were resistant to all antibiotics tested. The others showed susceptibility to colistin (76%), tigecycline (14%), trimethoprim/sulfamethoxazole (14%), gentamicin (14%), amikacin (10%), and/or tobramycin (10%)—for details, see the Supplementary Material (Table S1).

Each of the tested β-lactamase inhibitors significantly restored susceptiblity to aztreonam in the tested group of MBL bacteria. Table 1 presents descriptive data regarding the obtained MICs. Table 2 presents the results of the statistical analyses.

In three studied strains (one *K. pneumoniae* and two *E. coli*), none of the studied β-lactamase inhibitors restored aztreonam susceptibility. Moreover, in one strain (*K. pneumoniae*), only relebactam and vaborbactam restored the aztreonam susceptibility with absolutely no effect of avibactam.

## 3. Discussion

The most effective combination was aztreonam/avibactam, which restored aztreonam activity in more than 3/4 strains, while combinations with relebactam and vaborbactam did so in about 3/5 and almost 1/2 strains, respectively. However, we identified strains that regained aztreonam susceptibility only in combination with inhibitors other than avibactam. This suggests that we should not blindly (empirically) plan aztreonam/avibactam treatment in critical infections with Enterobacterale NDM. Such therapy should always be preceded by antibiotic susceptibility testing. The current problem is the lack of standardized methods and breakpoints from susceptibility standard development organizations (like EUCAST or CLSI). Until then, laboratories should rely on methods validated for scientific research, such as the method we used [12].

Fortunately, the Enterobacterales strains (one *K. pneumoniae* and two *E. coli*), in which none of the novel β-lactamase inhibitors had restored susceptibility to aztreonam, were susceptible to at least one tested antimicrobial. *E. coli* strains were susceptible to aminoglycosides and colistin. The *K. pneumoniae* strain was susceptible only to colistin. The use of these antibiotics should be carefully monitored because of the emergence of resistance to them. Additionally, aminoglycosides are not recommended for systemic monotherapy [13], and it is alarming that colistin resistance mechanisms (especially of the mcr type) are becoming more widespread [14].

Our results concerning aztreonam susceptibility restoration through avibactam in Enterobacterales NDM clinical strains were in accordance with several already published findings [12,15,16]. The high efficacy of the aztreonam/avibactam (as a concomitant administration of aztreonam and ceftazidime/avibactam) was also confirmed in patients with critical resistant infections through Enterobacterales NDM. Compared with other active antibiotics, aztreonam/avibactam is associated with lower clinical failure, shorter hospitalization, and lower mortality rates [17].

Biagi et al. [18,19] suggested that aztreonam in combination with relebactam or vaborbactam may be a viable treatment option for Enterobacterales NDM strains resistant to aztreonam. In our study, relebactam and vaborbactam restored aztreonam activity at lower rates than avibactam, which complies with other already published data [15,16]. Belati et al. [20] have suggested in an observational study that the concomitant administration of aztreonam and meropenem/vaborbactam is an effective therapy for patients with Enterobacterales NDM infection. There are no in vivo studies investigating the activity of the aztreonam/relebactam combination, but it may be predicted to be effective from data on other novel combinations.

Mecillinam is another beta-lactam stable against MBLs like aztreonam. It may therefore be another chance in the search for an appropriate therapy for infections caused by carbapenemase-producing Enterobacterales [7,8]. However, due to its pharmacokinetics, oral mecilinam (actually the prodrug pivmecilinam) is indicated only for use in urinary tract infections [21]. Intravenous mecillinam may be considered a treatment for bacteriaemia secondary to urinary tract infection but it is not registered widely for use [22]. For this reason, in our study, we analyzed only aztreonam, which has a wide range of applications. However, expanding research on the pharmacodynamic properties of amidopenicillins and searching for their new variants are interesting alternatives.

### Strengths and Limitations

This study analyzed a statistically sufficient sample (resulting from the calculated power of statistical tests—see Table 2) of Enterobacterales NDM, of which its resistance mechanism was molecularly confirmed by the national reference center. The main limitation is the genetic verification of the only selected resistance mechanisms (NDM, VIM, IMP, KPC, and OXA-48). Analyses of the entire genomes of the studied strains would provide a lot of valuable data, but would require many resources (financial and infrastructural).

## 4. Materials and Methods

In this retrospective study, we analyzed the antimicrobial resistance of clinical Enterobacterales NDM strains collected from the Central Teaching Hospital of Medical University of Lodz (Poland) in years 2021–2022. Ethical approval was not obtained, as studies involving only bacteria and no human data such as our study do not require ethical approval according to Polish law.

A total of 21 clinical Enterobacterales strains that produce NDM (and four co-producing OXA-48) carbapenemase and are simultaneously resistant to aztreonam, ceftazidime/avibactam, imipenem/relebactam, or meropenem/vaborbactam were investigated—17 *K. pneumoniae*, 3 *E. coli*, and 1 *C. freundii*. All bacteria were stored in Viabank storage beads (Medical Wire & Equipment, Corsham, UK) at a maximum of −80 °C for six months and regenerated on Columbia Agar with 5% sheep blood (Thermo Fisher Scientific, Waltham, MA, USA), 18–24 h at 37 °C. Phenotypic methods were used to assess the ability to produce carbapenemases, following the EUCAST guidelines [23]. Then, the presence of common carbapenem resistance mechanisms (KPC, OXA-48, NDM, and VIM) in tested strains was confirmed using the PCR method with the Polish National Reference Centre for Microbial Susceptibility (KORLD).

The minimal inhibitory concentrations (MICs) for aztreonam, ceftazidime/avibactam, imipenem/relebactam, and meropenem/vaborbactam were tested using the MIC Test Strips for AST (Liofilchem, Roseto degli Abruzzi, Italy) on Mueller-Hinton II agar plates (Thermo Fisher Scientific, Waltham, MA, USA). The MICs for aztreonam in combination with β-lactamase inhibitors (avibactam, relebactam, and vaborbactam) were tested using the gradient strip superposition method as described and validated by Emeraud et al. [12]. The concentrations of avibactam and relebactam were fixed at 4 mg/L, and that of vaborbactam, at 8 mg/L—this was due to the EUCAST recommendation regarding susceptibility testing for their currently available combinations with antibiotics. No standardization organization has yet issued official recommendations regarding aztreonam with inhibitors. Colistin susceptibility was assessed using the MICRONAUT MIC-Strip colistin assay (MERLIN Diagnostika, Bornheim-Hersel, Germany). Susceptibility to other antibiotics was assessed using an automated system VITEK 2 (bioMérieux, Marcy-l’Étoile, France); the assay included amoxicillin/clavulanate, piperacillin/tazobactam, cefuroxime, cefotaxime, ceftazidime, cefepime, imipenem, meropenem, amikacin, gentamicin, tobramycin, ciprofloxacin, tigecycline, and trimethoprim/sulfamethoxazole. The susceptibility interpretations were determined following EUCAST guidelines [13]. All determinations were performed in duplicate.

Statistical analyses were performed using Statistica 13 software (TIBCO Software, Palo Alto, CA, USA). The MIC values were presented as absolutes, and the changes in MICs in combination with β-lactamase inhibitors were calculated by dividing the value without by the values with the use of an inhibitor. The MIC value distribution was checked using the Shapiro–Wilk test. All data were transformed using the Box–Cox method, as the distributions of the variables were non-normal. After that, a t-test was used to measure effect size and the significance of the difference in comparing the groups. A *p*-value = 0.05 was considered the limit of statistical significance.

## 5. Conclusions

Overall, our study confirmed the previous suggestions that the combination of aztreonam/avibactam has the most significant antimicrobial potential for NDM-producing Enterobacterales isolates. However, the other combinations (with relebactam and vaborbactam) should not be rejected in advance because one clinical isolate was found in which only these drugs restored susceptibility to aztreonam—while avibactam did not. Further clinical studies are required to confirm the efficacy and safety of these antimicrobial combinations—especially concerning aztreonam/relebactam and aztreonam/vaborbactam. In addition, EUCAST and/or CLSI should, as soon as possible, develop methodology and standards for antimicrobial susceptibility testing for combinations of aztreonam with novel β-lactamase inhibitors.

## Figures and Tables

**Table 1:** Aztreonam (AZT) minimal inhibitory concentration (MIC) values and changes in Enterobacterales strains (*n* = 21) producing New Delhi Metallo-β-lactamases (NDM) depending on combination with novel β-lactam inhibitor—avibactam (AVI), relebactam (REL), or vaborbactam (VAB).

	MIC Range [mg/L]	MIC_90_ [mg/L]	MIC_50_ [mg/L]	MIC Change Range vs. AZT	% of AZT Susceptiblity Restoration
AZT	8–>256	>256	96		
AZT/AVI	0.047–48	8	0.19	0–11	80.95
AZT/REL	0.19–128	8	4	0.7–4	61.90
AZT/VAB	0.094–48	16	6	1.4–9.4	47.62

**Table 2:** Descriptive statistic and t-test analysis results for comparison of Box–Cox minimal inhibitory concentrations (MICs) data of Enterobacterales strains (*n* = 21) producing New Delhi Metallo-β-lactamases (NDM) for aztreonam (AZT) versus aztreonam/avibactam (AZT/AVI), aztreonam/relebactam (AZT/REL), and aztreonam/vaborbactam (AZT/VAB).

	Box–Cox Lambda	Mean	Standard Deviation	Mean Difference	t	df	*p*	CI95%	Power
AZT	0.159	6.26	1.78						
AZT/AVI	−0.461	−2.05	2.14	8.31	15.83	20	<0.001	7.21–9.40	1
AZT/REL	−0.030	0.95	1.46	5.30	15.85	20	<0.001	4.61–6.00	1
AZT/VAB	0.147	1.50	1.81	4.76	12.80	20	<0.001	3.98–5.53	1

## Data Availability

The data presented in this study are available in the Supplementary Materials (Table S1).

## Supplementary Materials

The following supporting information can be downloaded at https://www.mdpi.com/article/10.3390/ph17030383/s1: Table S1: The data presented in the study.
