# Supplementary material for: Aztreonam Combinations with Avibactam, Relebactam, and Vaborbactam as Treatment for New Delhi Metallo-β-Lactamase-Producing Enterobacterales Infections—In Vitro Susceptibility Testing
Source: Pharmaceuticals (Basel). 2024 Mar 17;17(3):383. doi: 10.3390/ph17030383 (PMC10975290; doi:10.3390/ph17030383)
Supplement: Supplementary file 1 [file pharmaceuticals-17-00383-s001.zip › pharmaceuticals-2877313-supplementary.pdf]

Table S1. The data presented in the study.

No	Species	Genes detected		Aztreonam		Aztreonam/ avibactam			Aztreonam/ relebactam			Aztreonam/ vaborbactam			Colistin		Other antibiotics with susceptibility
		NDM	OXA-48	MIC [mg/L]	Int.	MIC [mg/L]	Int.	Change	MIC [mg/L]	Int.	Change	MIC [mg/L]	Int.	Change	MIC [mg/L]	Int.	
1	*K. pneum.*	+		8	R	0.19	S	5.4	0.25	S	5.0	0.38	S	4.4	0.25	S	
2	*K. pneum.*	+		38	R	38	R	0,0	0.25	S	7.2	0.38	S	6.6	0.25	S	TIG
3	*K. pneum.*	+	+	48	R	0.125	S	8.6	3	I	4.0	1	S	5.6	1	S	
4	*K. pneum.*	+		32	R	0.094	S	8.4	3	I	3.4	1.5	I	4.4	0.063	S	
5	*K. pneum.*	+		48	R	0.125	S	8.6	0.5	S	6.6	2	I	4.6	1	S	
6	*K. pneum.*	+		32	R	0.094	S	8.4	1.5	I	4.4	2	I	4.0	0.25	S	
7	*K. pneum.*	+		32	R	0.19	S	7.4	0.19	S	7.4	3	I	3.4	0.25	S	
8	*K. pneum.*	+		256	R	0.19	S	10.4	2	I	7.0	4	I	6.0	16	R	
9	*K. pneum.*	+	+	32	R	0.25	S	7.0	4	I	3.0	6	R	2.4	16	R	TRI/SME
10	*K. pneum.*	+	+	96	R	0.25	S	8.6	4	I	4.6	6	R	4.0	32	R	
11	*K. pneum.*	+	+	128	R	0.25	S	9.0	4	I	5.0	6	R	4.4	1	S	GEN, TRI/SME
12	*K. pneum.*	+		32	R	0.19	S	7.4	6	R	2.4	6	R	2.4	0.125	S	TRI/SME
13	*K. pneum.*	+		>256	R	0.19	S	10.4	6	R	5.4	8	R	5.0	32	R	
14	*K. pneum.*	+		>256	R	0.25	S	10.0	8	R	5.0	8	R	5.0	0.25	S	TIG
15	*K. pneum.*	+		128	R	0.38	S	8.4	8	R	4.0	8	R	4.0	16	R	TIG
16	*K. pneum.*	+		96	R	0.25	S	8.6	8	R	3.6	16	R	2.6	0.125	S	
17	*K. pneum.*	+		128	R	48	R	1.4	128	R	0.0	48	R	1.4	0.125	S	
18	*E. coli*	+		96	R	0.047	S	11.0	0.75	S	7.0	0.38	S	8.0	0.125	S	
19	*E. coli*	+		96	R	8	R	3.6	6	R	4.0	16	R	2.6	0.063	S	AMI, GEN, TOB
20	*E. coli*	+		>256	R	6	R	5.4	8	R	5.0	24	R	3.4	0.125	S	AMI, GEN, TOB
21	*C. freundii*	+		64	R	0.125	S	9.0	1.5	I	5.4	0.094	S	9.4	0.5	S	

Int. – Interpretation; *K. pneum.* – *Klebsiella pneumoniae*; *E. coli* – *Escherichia coli*; *C. freundii* – *Citrobacter freundii*; NDM – New Delhi metallo-β-lactamase; OXA-48 – oxacillinase-48; MIC – minimum inhibitory concentration; S – susceptible, I – susceptible, increased exposure; R – resistant; TIG – tigecycline; TRI/SME – trimethoprim/ sulfamethoxazole; GEN – gentamicin; AMI – amikacin; TOB – tobramycin
